# Linalool, a *Piper aduncum* essential oil component, has selective activity against *Trypanosoma cruzi* trypomastigote forms at 4°C

**DOI:** 10.1590/0074-02760160361

**Published:** 2017-02

**Authors:** Luz Helena Villamizar, Maria das Graças Cardoso, Juliana de Andrade, Maria Luisa Teixeira, Maurilio José Soares

**Affiliations:** 1 Fundação Oswaldo Cruz-Fiocruz, Instituto Carlos Chagas, Laboratório de Biologia Celular, Curitiba, PR, Brasil; 2 Universidade Federal de Lavras, Departamento de Química, Lavras, MG, Brasil

**Keywords:** essential oil, linalool, Piper aduncum, Trypanosoma cruzi, trypanocidal activity

## Abstract

**BACKGROUND:**

Recent studies showed that essential oils from different pepper species (*Piper* spp.) have promising leishmanicidal and trypanocidal activities.

**OBJECTIVES:**

In search for natural compounds against *Trypanosoma cruzi*, different forms of the parasite were incubated for 24 h at 28ºC or 4ºC with *Piper aduncum* essential oil (*Pa*EO) or its main constituents linalool and nerolidol.

**METHODS:**

*Pa*EO chemical composition was obtained by GC-MS. Drug activity assays were based on cell counting, MTT data or infection index values. The effect of *Pa*EO on the *T. cruzi* cell cycle and mitochondrial membrane potential was evaluated by flow cytometry.

**FINDINGS:**

*Pa*EO was effective against cell-derived (IC_50_/24 h: 2.8 μg/mL) and metacyclic (IC_50_/24 h: 12.1 μg/mL) trypomastigotes, as well as intracellular amastigotes (IC_50_/24 h: 9 μg/mL). At 4ºC - the temperature of red blood cells (RBCs) storage in blood banks - cell-derived trypomastigotes were more sensitive to *Pa*EO (IC_50_/24 h = 3.8 μg/mL) than to gentian violet (IC_50_/24 h = 24.7 mg/mL). Cytotoxicity assays using Vero cells (37ºC) and RBCs (4ºC) showed that *Pa*EO has increased selectivity for cell-derived trypomastigotes. Flow cytometry analysis showed that *Pa*EO does not affect the cell cycle of *T. cruzi* epimastigotes, but decreases their mitochondrial membrane potential. GC-MS data identified nerolidol and linalool as major components of *P*aEO, and linalool had trypanocidal effect (IC_50_/24 h: 306 ng/mL) at 4ºC.

**MAIN CONCLUSION:**

The trypanocidal effect of *Pa*EO is likely due to the presence of linalool, which may represent an interesting candidate for use in the treatment of potentially contaminated RBCs bags at low temperature.

The protozoan *Trypanosoma cruzi* is the etiologic agent of Chagas disease, a neglected tropical illness that is endemic in 21 Latin American countries (WHO 2016), and has spread to five continents, mainly due to immigration (Schmunis & Yadon 2010). Chagas disease has no effective treatment and affects about 7-8 million people worldwide (WHO 2016), with about five million of those in Brazil (Salomon 2012). *T. cruzi* transmission routes include contact with infected blood-sucking insects (Rassi et al. 2009), blood transfusion (Assal & Corbi 2011), vertical transfer from mother to fetus (Jackson et al. 2009), organ and tissue transplantation (Fishman & Rubin 1998, Schmunis & Yadon 2010), contact with infected conjunctiva or oral mucosa (Giddings et al. 2006), and laboratory accidents (Herwaldt 2001).

Benznidazole (BZ) is the drug commonly used to treat individuals with Chagas disease (Rajão et al. 2014). Treatment with BZ has approximately 80% efficacy in the acute phase and 20% in the chronic phase of the disease (Rassi et al. 2009). The side effects of BZ - which include allergic dermatitis, paresthesia, thrombocytopenia and leucopenia - lead to treatment discontinuation in 7-13% of cases (Bern 2011). Despite decades of efforts to obtain less toxic, easily accessible (and relatively inexpensive) drugs against Chagas disease, no effective molecule or compound, whether natural or synthetic, has yet been identified that could replace BZ in the clinic, to improve the quality of life of affected patients (Romanha et al. 2010).

From the natural products currently tested against *T. cruzi* the most studied alternatives are of plant origin (Coura & de Castro 2002). The trypanocidal activity of approximately 400 plants species (over 100 plant families) has been analysed in the last two decades (Izumi et al. 2012). Nevertheless, relatively few reports have assessed the anti-trypanocidal activity of essential oils (EOs) (Alviano et al. 2012), volatile and aromatic compounds produced (as secondary metabolites) by all plant organs. In plants, EOs function as antiseptics and biocides, protecting not only against pathogenic microorganisms but also against herbivores. Due to their pleasant odor and low toxicity profile, several EOs are widely used in perfumes, food products and herbal medicines (Bakkali et al. 2008). EOs, or their major constituents, represent promising candidates for the development of drugs against *T. cruzi*. A number of different EOs are active against *T. cruzi* and not appreciably toxic to mammalian cells (Borges et al. 2012).

Peppers (*Piper* spp., Piperaceae) form a large genus of plants (> 700 species) widespread in tropical and subtropical regions of the world, and used in traditional medicine as analgesic, antiseptic and antimicrobial agents (Orjala et al. 1994, Xu & Li 2011). Whole extracts or purified molecules derived from a variety of pepper species have activity against *Leishmania* and *Trypanosoma*, usually in the 10-20 µg/mL range (Lopes et al. 2008, Regasini et al. 2009, Garcia et al. 2013). Pepper EOs are also active against trypanosomatid parasites. *Piper auritum* EO inhibits the proliferation of promastigotes of *Leishmania major*, *L. mexicana*, *L. braziliensis* and *L. donovani*, with IC_50_ values (for 72 h treatments) between 12.8 and 63.3 µg/mL (Monzote et al. 2010). Also *P. hispidum* EO showed promising results (high antileishmanial activity with low cytotoxicity, and a safety index of eight) when treating *L. amazonensis*-infected macrophages (Houël et al. 2015). The EO of *P. cubeba* was not active against *L. amazonensis*, but was effective against *T. cruzi*, with IC_50_ values of 45.5 and 87.9 µg/mL, for trypomastigote and amastigote forms, respectively (Esperandim et al. 2013).

*P. aduncum* (common names: ‘aperta-ruão’, ‘jaborandi-falso’, ‘pimento-de-macaco’ and ‘matico’) is a tropical bush typical from Central and South America. In Brazil it is found naturally in the Amazon and Atlantic Forests. *P. aduncum* extracts inhibits the proliferation of *T. cruzi* epimastigotes in vitro, with IC_50_ concentrations lower than 20 µg/mL (Flores et al. 2009). Also, five chromenes isolated from *P. aduncum* have anti-proliferative activity against *T. cruzi* epimastigotes, with IC_50_ values of 2.82 µM, after 72 h of treatment (Batista Jr et al. 2008), suggesting that the anti-trypanocidal activity of this plant should be analysed further. Despite the trypanocidal activity of *P. aduncum* extracts, no studies have yet tested the effect of *P. aduncum* EOs against *T. cruzi*.

Here, we analysed the trypanocidal effect of *P. aduncum* EO (*Pa*EO) against different developmental forms of *T. cruzi*, in bioassays for 24 h. Gas chromatography-mass spectrometry (GC-MS) data identified nerolidol and linalool as the major components of this essential oil, and linalool had strong trypanocidal effect (IC_50_/24 h: 306 ng/mL) at 4ºC. The promising trypanocidal effect of *Pa*EO is likely due to the presence of linalool, which may be used as a lead for further drug development.

## MATERIALS AND METHODS

*Vero cells* - Vero cells (ATCC CCL-81) were grown in RPMI-1640 medium with L-glutamine (Sigma Aldrich, St. Louis, MO, USA), supplemented with 5% fetal calf serum (FCS; Cultilab, Campinas, SP, Brazil), at 37ºC, and in a humidified 5% CO_2_ atmosphere. For seeding, cell monolayers were washed twice with PBS (pH 7.2), trypsinized and collected by centrifugation at 100 *g* for 2 min.

*Parasites* - In this work, we used the *T. cruzi* clone Dm28c. Epimastigote forms were kept at 28ºC in LIT medium supplemented with 10% inactivated FCS, with passages at every three days. For the experiments, parasites obtained from 72-h cultures were used.

To obtain cell-derived trypomastigotes, Vero cells were incubated with cell-derived trypomastigotes (1:10 ratio of cells to parasites) in 75 cm^2^ culture flasks containing 8 mL DMEM. After 4 h of interaction, non-internalised parasites were removed by rinsing with phosphate-buffered saline (PBS), new medium was added, and then changed to fresh medium every 24 h. After 96 h of infection, trypomastigotes released into the supernatant were collected by centrifugation at 3000 *g* for 10 min.

To obtain metacyclic trypomastigotes, culture epimastigotes in late logarithmic growth phase (five days) were subjected to nutritional stress as described previously (Contreras et al. 1985). Culture epimastigotes (5-7 x 10^7^ cells/mL) were collected by centrifugation at 7000 *g* for 5 min at 10ºC, and resuspended in TAU medium (Triatomine Artificial Urine: 190 mM NaCl, 17 mM KCl, 2 mM MgCl_2_, 2 mM CaCl_2_, 8 mM phosphate buffer pH 6.0), at a concentration of 5 x 10^8^ cells/mL. After incubation for 2 h at 28ºC (nutritional stress), parasites were transferred to 25 cm^2^ flasks containing 5 mL TAU3AAG medium (TAU supplemented with 10 mM L-proline, 50 mM sodium glutamate, 2 mM sodium aspartate and 10 mM glucose), at a final concentration of 5 x 10^6^ cells/mL. After 72 h, metacyclic trypomastigotes in the supernatant (~80% parasites) were collected by centrifugation and purified by passage through a DEAE-cellulose affinity column, equilibrated in PSG buffer (47.47 mM Na_2_HPO_4_, 2.5 mM NaH_2_PO_4_.H_2_0, 37.76 mM NaCl, 55.5 mM glucose).

*P. aduncum essential oil (PaEO) purification and chemical analysis* - *P. aduncum* L. (matico) leaves were collected on March 2013 in the morning, with no rain, at the Medicinal Plants Garden of the Universidade Federal de Lavras (UFLA, MG, Brazil). *P. aduncum* EO was obtained by distillation in a Clevenger equipment, at the Department of Chemistry, Federal University of Lavras. Prior to use in experiments, *Pa*EO was diluted to 100 mg/mL in DMSO (*Pa*EO stock solution). Final DMSO concentrations in activity assays did not exceed 0.5%. Both undiluted and diluted (stock) *Pa*EO were kept at 4ºC, and in the dark.

*Pa*EO chemical analysis was performed in the Department of Chemistry of UFLA, in a GC-17A Shimadzu gas chromatograph coupled to a QP 5000 Shimadzu mass spectrometer, with a selective detector. The column used was of fused silica/bound type (DB5, 30m x 0.25mm), with helium (1 mL/min) as the mobile-phase gas. The following analysis conditions were used: injection at 220ºC, detection at 240ºC; oven temperature between 40ºC and 240ºC, with addition of 3ºC/min; initial column pressure of 100.2 kPa; 1:10 split ratio and injected volume of 1 µL (solutions at 1% v/v) in dichloromethane. Mass spectra of each compound were compared with the Wiley 229 library database, and with the tabulated Kovats index.

*Single drug activity assays on T. cruzi developmental forms* - All experiments were performed in biological and technical triplicates. Incubations were performed for 24 h in all bioassays. The absorbance of untreated cells in culture media containing 0.5% DMSO (control) was used as 100% cell viability, and the percentage of dead cells in each treatment was estimated by comparison with the untreated control.

For all assays, IC_50_/24 h values (based on cell counting, MTT data or infection index values) and dose-response curves were generated using the CompuSyn software (ComboSyn Inc., Paramus, NJ, USA), and statistical analysis was performed in Excel. For all assays, the fraction of affected cells (Fa) was calculated relative to the untreated control (treated/untreated ratio).

To test the activity of *Pa*EO on epimastigote forms, these cells were seeded into 96-well plates (5 x 10^6^ cells/well) in LIT medium, and treated for 24 h at 28ºC with *Pa*EO at the final concentrations of 9, 18, 37, 75, 150 and 300 µg/mL. As a control, BZ (at 6.25, 12.5, 25 or 50 µg/mL) or LIT medium with 0.5% DMSO (negative control) were used. Cell viability was assessed by the MTT assay (see “MTT assay”).

*Pa*EO activity assays with cell-derived trypomastigotes were performed at 28ºC and 4ºC. Cell-derived trypomastigotes (5 x 10^7^ cells/well) were seeded in 96-well plates with DMEM, and treated with *Pa*EO or BZ at the final concentrations 1, 10, 50 and 100 µg/mL, or with gentian violet (1 and 25 µM; for 4ºC experiments only). Untreated cells were used as a negative control. For treatments at 28ºC, plates were incubated at this temperature for 24 h. Then, each well was diluted 1:10 with 10% formaldehyde in PBS and cells were counted in a hemocytometer. For treatment at 4ºC, plates were incubated at this temperature for 24 h and then subjected to the MTT assay (see “MTT assay”). Due to the small size of trypomastigotes, the MTT reagent was used at the concentration of 1 mg/mL (2.5 fold less than that used for epimastigotes), which improved the correlation between the number of viable cells and the optical density (not shown). The major constituents of *P. aduncum* EO - linalool and nerolidol (both from Sigma, St. Louis, MO, USA) - were also tested (at the concentrations of 100, 250, 500 or 1000 ng/mL) against *T. cruzi* cell-derived trypomastigotes at 4ºC, as described above.

To test *Pa*EO activity on metacyclic trypomastigotes, these cells were plated in 96-well plates (5 x 10^6^ cells/well) with TAU3AAG medium, and treated with EO at the final concentrations of 1, 10, 50 or 100 µg/mL, for 24 h at 28ºC. Then, each well was diluted at 1:10 with 10% formaldehyde in PBS, and cells were counted in a hemocytometer.

To test *Pa*EO activity against intracellular amastigotes, Vero cells were seeded in 24-well plates (2 x 10^4^ cells/well) in DMEM (Sigma-Aldrich), and allowed to adhere for 24 h. Then, cell-derived trypomastigotes were added to each well (1:10 ratio of cells to parasites), incubated for 3 h, and non-internalised parasites were removed by washing with PBS. Infected cultures were incubated for 24 h (at 37ºC, 5% CO_2_), and then the culture medium was replaced with RPMI-1640 containing *Pa*EO or BZ at final concentrations between 1 and 100 µg/mL (total volume of 1 mL/well), and plates were incubated for 24 h, at 37ºC (5% CO_2_). Treated cells were fixed with methanol, stained with Giemsa, and the inhibitory effect on intracellular amastigotes was estimated by counting (a) the number of infected cells, and (b) the number of amastigotes per cell, in 100 cells/wells, from random light microscopy images. These data were used to calculate the infection index (II) for each tested concentration (II = % infected cells x number of amastigotes per cell).

*MTT assay* - After drug treatments, MTT solution (10 mg/mL in PBS) was added to each well for a final concentration of 2.5 mg/mL (or 1 mg/mL for trypomastigotes). Plates were incubated for 3 h at 28ºC in the dark, centrifuged for 10 min at 475 *g* and the supernatant was removed (by quick plate reversal). Then, 20 µL SDS was added to each well, followed by incubation for 1 h at 28ºC, after which 80 µL DMSO was added to each well and incubated for a further 1 h at 28ºC. Finally, the residual material was removed with the aid of a toothpick, and sample absorbance (at 550 nm) was read in an EL800 microplate reader (Biotek, Winooski, VT, USA). Dose-response curves were produced using the CompuSyn software, which was also used to calculate IC_50_/24 values.

*Cytotoxicity* - Vero cells were seeded in 96-well plates (2 x 10^4^ cells/well) with RPMI-1640 medium and cultivated for 24 h at 37ºC (5% CO_2_). Cells were incubated for a further 24 h in the presence of *Pa*EO (9, 18, 37, 75, 150 or 300 µg/mL), linalool/nerolidol (30, 60, 125, 250 or 500 ng/mL), BZ (1, 10, 100 or 1000 µg/mL) or gentian violet (100, 500 or 1000 µg/mL). Then, plates were subjected to the MTT assay as described above (see “MTT assay”), and MTT data were used to calculate 50% cytotoxicity (CC_50_/24 h) values. Plates were examined in an inverted microscope every 12 h, to evaluate monolayer integrity (confluence and adhesion).

To analyse cytotoxicity on red blood cells (RBCs) at 4ºC, human erythrocytes were obtained from 10-mL blood samples (O+), from a healthy volunteer donor, as previously described (Izumi et al. 2012). After collection, blood was defibrillated and then washed with a sterile ‘saline-glucose’ solution (0.85% NaCl/5% glucose). The final pellet was diluted in saline-glucose and centrifuged for 5 min at 1400 *g*. The supernatant was discarded and the pellet of RBCs was diluted in saline-glucose, for a final concentration of 6%. Then, RBCs were seeded in 96-well plates (3% RBCs/well) and treated with EO (0.1, 0.2, 0.4, 0.8, 1.6 or 3.2 mg/mL), for 5 h, at 4ºC. Negative control samples represented untreated RBCs diluted in saline-glucose. After treatment, plates were centrifuged at 1027 *g* for 2 min, and 100 µL of supernatant from each well were transferred to a new plate and analysed (at 550 nm) in an EL800 microplate reader (Biotek, Winooski, VT, USA).

Cytotoxicity (CC_50_) values were calculated using Excel, and selectivity index (SI) values were calculated as the ratio between the CC_50_/24 h (for Vero cells or RBCs) and IC_50_/24 h values.

*Flow cytometry* - For flow cytometry analysis, epimastigotes were seeded into 96-well plates (5 x 10^6^ cells/well) in LIT medium and treated with *Pa*EO at a concentration corresponding to the IC_50_/24 h, for 24 h. Negative control cells were kept untreated. Then, cells were washed twice in PBS (with centrifugation at 7000 *g* for 2 min) and transferred to plastic flow cytometry tubes (300 µL/tube).

To evaluate the effect of *Pa*EO on the *T. cruzi* cell cycle, 1 mL of 10 µg/mL propidium iodide diluted in 5% NP40, with 20 µg/mL RNAse (Qubit RNA HS Assay Kit, ThermoFisher Scientific, Waltham, MA, USA) was added to each tube, and samples were incubated for 20 min at 28ºC.

To evaluate the effect of *Pa*EO on *T. cruzi* mitochondrial membrane potential, rhodamine 123 (final concentration 10 µg/mL) was added to cultures after *Pa*EO treatment, and cells were incubated for 20 min at 28ºC, before PBS washing and transfer to flow cytometry tubes. Culture treatment with 10 µM CCCP (carbonyl cyanide 3-clorophenylhydrazone, Sigma Aldrich, St. Louis, MO, USA) after the rhodamine incubation was used as a positive control.

Flow cytometry samples were analysed in a FACS Canto II (Becton-Dickinson, San Jose, CA, USA) flow cytometer (20,000 events/sample; 488 nm excitation and 585/42 nm emission for propidium iodide; 488 excitation and 530/30 emission for rhodamine 123), and data analysis was performed in FlowJo (Treestar Software, Ashland, OR, USA). Statistical analysis was performed by one-way analysis of variance (ANOVA), using GraphPad Prism 5.01 software.

## RESULTS

**Fig. 1:**
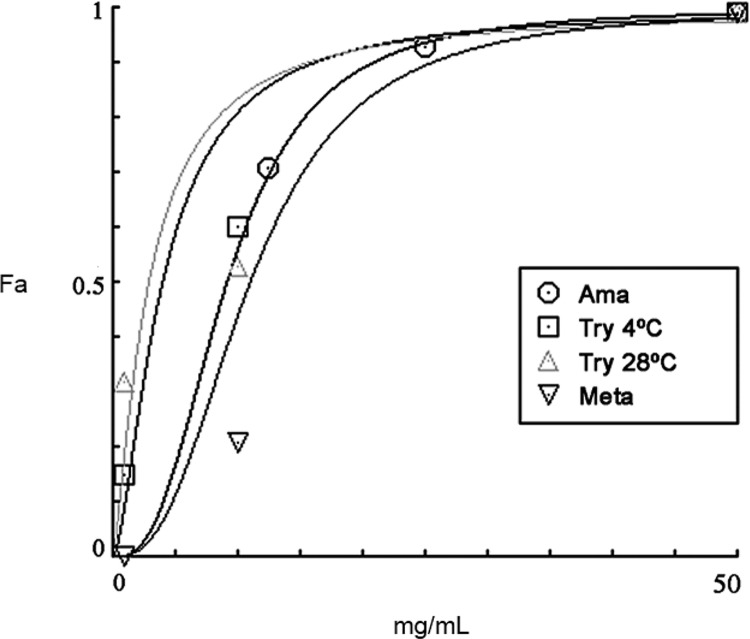
: effect of *Piper aduncum* essential oil (*Pa*EO) on *Trypanosoma cruzi*. Dose-response curves showing the inhibitory effect of *Pa*EO on *T. cruzi* metacyclic trypomastigotes (meta), cell-derived trypomastigotes (try) and intracellular amastigotes (ama). Fa = Fraction of affected cells (ratio to untreated control).

*Cell-derived T. cruzi trypomastigotes are sensitive to PaEO* - The EO of *P. aduncum* - a pepper species that has already yielded anti-trypanocidal extracts and molecules - had not been tested for its biological activity against *T. cruzi*. Thus, we tested the purified *Pa*EO against different *T. cruzi* developmental forms, namely epimastigotes (axenically grown insect forms), cell-derived amastigotes and trypomastigotes (produced in monolayers of mammalian host cells) and infective metacyclic trypomastigotes (differentiated, by metacyclogenesis in vitro, from epimastigotes) (Fig. 1). As a reference drug, we used BZ, the current first-line for Chagas disease.

Cell-derived trypomastigotes were more sensitive to *Pa*EO (IC_50_/24 h at 28ºC = 2.8 µg/mL) than to BZ (IC_50_/24 h = 16.1 µg/mL) (Table I). Metacyclic trypomastigotes were also sensitive to *P. aduncum* EO, with IC_50_/24 h values for *Pa*EO and BZ of 12.1 µg/mL and 0.3 µg/mL, respectively.

**TABLE I:** Inhibitory effect (IC50/24 h) and selectivity index (SI) of *Piper aduncum* essential oil (*Pa*EO) for the treatment of different *Trypanosoma cruzi* developmental forms, compared with benznidazole (BZ)

	IC_50_/24 h (mg/mL)		Selectivity index (SI)	

	*Pa*EO	BZ	*Pa*EO^a^	BZ^b^
Cell-derived trypomastigotes (28ºC)	2.8	16.1	15.3	63.4
Cell-derived trypomastigotes (4ºC)	3.8	ND	11.3	ND
Metacyclic trypomastigotes (28ºC)	12.1	0.3	3.5	3,646.4
Epimastigotes (28ºC)	84.7	14.7	0.5	69.4
Amastigotes (37ºC)	9	0.8	4.7	1,276.2

*a*: SI = CC_50_ *Pa*EO on Vero cells (42.8 µg/mL) / IC_50_ *Pa*EO; *b*: SI = CC_50_ BZ on Vero cells (1021 µg/mL) / IC_50_ BZ; ND: not done.

*T. cruzi* epimastigotes were incubated for 24 h at 28ºC with different concentrations of *Pa*EO or BZ, and dose-response curves estimated the IC_50_/24 h values as 84.7 µg/mL for *Pa*EO and 14.7 µg/mL (45 µM) for BZ (Table I).

Blood transfusion is an important route for Chagas disease transmission, due to the presence of infective trypomastigotes in blood from infected donors. Thus, we tested the sensitivity of cell-derived trypomastigotes to *Pa*EO at 4ºC, the temperature used for red blood storage in blood banks. Incubation at 4ºC led to inactivation of BZ, as evidenced by the presence of clumps of crystalline material in the medium (likely representing BZ agglutination), and by the absence of trypanosome morphological damage or motility loss, as assessed by light microscopy observation (not shown). Therefore, for the experiments at 4ºC we used gentian violet as a control, since this drug can be used to treat blood potentially infected with Chagas disease (Ramirez et al. 1995). Importantly, cell-derived trypomastigotes were 6,500 times more sensitive to *P. aduncum* EO (IC_50_/24 h = 3.8 µg/mL) than to gentian violet (IC_50_/24 h = 24,700 mg/mL).

To estimate *Pa*EO cytotoxicity, we performed cytotoxicity assays in uninfected Vero cells at 37ºC (Table II), and also in human erythrocytes (RBCs) at 4ºC (to mimic red blood cell storage conditions in blood banks). *Pa*EO was more cytotoxic to Vero cells than BZ, with a CC_50_/24 h of 42.8 µg/mL, compared with 1,021 mg/mL for BZ. At the treatment temperature of 4ºC, gentian violet also exhibited lower cytotoxicity to RBCs (CC_50_/24 h = 71.4 mg/mL) than *Pa*EO (CC_5_/24 h = 351.6 µg/mL).

**TABLE II:** Inhibitory effect (IC50/24 h, in µg/mL) and cytotoxicity (CC50/24 h, in µg/mL) of *Piper aduncum* essential oil (*Pa*EO), gentian violet and linalool for *Trypanosoma cruzi* cell-derived trypomastigotes, red blood cells (RBCs, at 4ºC) and Vero cells (at 37ºC)

	*T. cruzi* IC_50_/24 h	RBCs CC_50_/24 h	Selectivity index (SI) - RBCs	Vero cells CC_50_/24 h	Selectivity index (SI) - Vero cells
*Pa*EO	3.8	351.6	92.5	42.8	11.2
Linalool	0.31	7,341.6	23,990	0.87	2.7
Gentian Violet	24,700	> 70,000	> 2.8	71,400	2.9

Selectivity index (SI) analysis - representing the ratio between IC_50_/24 h and CC_50_/24 h values - indicates that *Pa*EO is particularly selective towards cell-derived *T. cruzi* trypomastigotes (SI = 15.3 and 11.3, for treatments with Vero cells at 28ºC and 4ºC, respectively; Table I). Importantly, at the treatment temperature of 4ºC, *Pa*EO was more selective towards this form of the parasite than gentian violet (SI = 2.9).

*PaEO inhibits effectively the intracellular survival/replication of T. cruzi amastigotes* - To test the effect of *Pa*EO on amastigotes, Vero cells were infected with trypomastigotes and, after 24 h (when trypomastigotes had differentiated into amastigotes), the infected cultures were incubated with different concentrations of *Pa*EO (or BZ, as a reference). *Pa*EO at the concentration of 12.5 µg/mL decreased the *T. cruzi* amastigote infection index by 71.5%, similarly to 10 µg/mL BZ (81.3% decrease; Table III), showing that *Pa*EO is as effective as the standard drug used for Chagas disease treatment, at this concentration. However, BZ was much more efficient than *Pa*EO at inhibiting intracellular amastigote replication and survival, with an IC_50_/24 h (calculated from infection index data) of 0.8 µg/mL, compared with 9 µg/mL for *Pa*EO (Table I). Also, strong cytotoxic effects occurred after treatment with 100 µg/mL *Pa*EO (data not shown).

**TABLE III:** Effect of 24 h-treatment with *Piper aduncum* essential oil (*Pa*EO) on *Trypanosoma cruzi* intracellular amastigotes in Vero cells, compared with benznidazole (BZ)

	Concentration (µg/mL)	Infected cells (%)	Amastigotes per cell	Infection index (II)	II decrease
*Pa*EO	0	79.1	7.1	562.1	0
	12.5	54.6	2.9	160.1	71.5
	50	32.5	0.4	12.7	97.7
	100	0	0	0	100
BZ	0	87.3	11.5	1003.7	0
	10	74.9	2.5	187.2	81.3
	50	0.4	0.9	0.3	99.9

To examine the mechanism of action of *Pa*EO on *T. cruzi*, epimastigotes were incubated for 24 h at 28ºC with the EO and then subjected to cell cycle analysis by labeling with propidium iodide, or were labelled with Rhodamine-123, to analyse the effect of *Pa*EO on the mitochondrial membrane potential. Although epimastigotes do not have direct medical importance, this life cycle form is ideal for in vitro analysis, because they can be cultivated easily in axenic medium.

**Fig. 2:**
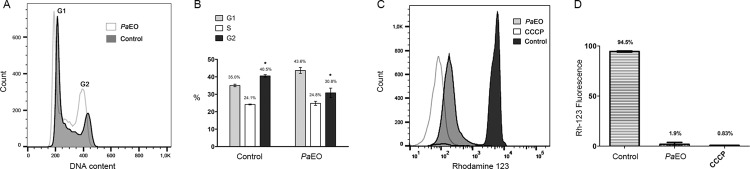
: effect of *Piper aduncum* essential oil (*Pa*EO) on *Trypanosoma cruzi* epimastigotes, as analysed by flow cytometry. (A-B) Cell cycle analysis. Epimastigotes were treated for 24 h at 28°C with the IC50/24 h value of *Pa*EO and then incubated with propidium iodide (PI). (A) Histograms of the PI-stained cell populations; (B) mean number of cells in each cell cycle stage. *: p < 0.05. (C-D) Mitochondrial membrane potential analysis. Epimastigotes were kept for 24 h at 28°C with the IC50/2 h value of *Pa*EO and then incubated with Rhodamine-123. CCCP (carbonyl cyanide 3-clorophenylhydrazone): positive control. (C) Histograms of the Rhodamine-123-stained populations; (D) percentage of labelled cells after each treatment.

Treatment of epimastigotes with *P*aEO at the IC_50_/24 h did not alter significantly the number of cells in the G1 phase of the cell cycle, but there was a significant decrease in the number of cells in G2 (Fig. 2A-B). Labeling with rhodamine-123 showed that the *Pa*EO decreased the mitochondrial membrane potential of nearly 98% of the tested epimastigotes (Fig. 2C-D), indicating a possible target of this compound.

Chemical composition analysis by gas chromatography/mass spectrometry (GC-MS) showed that the main constituents of the *Pa*EO used in this study are nerolidol (25.22%) and linalool (13.42%) (Table IV). Aside from these two main constituents, *Pa*EO contained several minor components (each accounting for ~1-5% of the total area; Table IV). Therefore, we tested the activities of linalool and nerolidol against cell-derived trypomastigotes at 4ºC (for 24 h), because the activity of *Pa*EO against this developmental form appeared to be particularly promising (Table I). Linalool had clear trypanocidal activity, with an IC_50_/24 h of 306 ng/mL at 4ºC (Table II), indicating that this compound is approximately 52 times more effective against cell-derived trypanosomes than BZ at 28ºC (Table I). Cytotoxicity tests on Vero cells (at 4ºC) demonstrated that linalool was cytotoxic, however, with a CC_50_/24 h of 823.6 ng/mL, which resulted in a selectivity index (SI) for the treatment of cell-derived trypomastigotes at 4ºC of 2.69 (Table II).

**TABLE IV:** Chemical composition of *Piper aduncum* essential oil (*Pa*EO), as assessed by gas chromatography/mass spectrometry (GC/MS)

Constituent	Retention time	Area	Area (%)
Nerolidol	31.13	18000000	25.22
Linalool	11.83	9336350	13.42
Spatulenol	31.77	4374871	6.29
β-ocimene	9.76	3237239	4.65
2-cycloexen-1-ol	25.48	3092676	4.44
Germacrene D	27.99	2804545	4.03
β-chamigrene	28.59	2788502	4.01
E-β-farnesene	26.95	2509366	3.61
γ-cadinene	29.37	2439762	3.51
α-epi-muurolol	34.25	1893537	2.72
α-bisabolol oxide	34.75	1867181	2.68
α-ocimene	9.36	1807180	2.6
Turmerone	32.49	1437434	2.07
Caryophyllene oxide	31.98	1248458	1.79
α-copaene	23.65	1169184	1.68
Viridiflorol	33.04	1117593	1.61
β-pinene	7.5	988059	1.42
α-cadinol	34.43	975559	1.4
α-cubebene	24.16	884691	1.27
δ-cadineme	29.5	854201	1.23
α-pinene	6.2	680357	0.98
Safrole	19.94	640257	0.92

In contrast to linalool, nerolidol had no appreciable trypanocidal activity against cell-derived trypomastigotes (4ºC, 24 h) at concentrations between 100 ng/mL and 1 µg/mL, as assessed by light microscopy observation and MTT assays (data not shown). Also, nerolidol did not affect the integrity of Vero cell monolayers, after treatment for 24 h with the highest concentration used (1 µg/mL; not shown). These data indicate that the trypanocidal effect of *Pa*EO was due to linalool.

## DISCUSSION

The microbicidal activity of EOs - against fungi, bacteria and viruses - is used primarily as a defense to ensure plant survival (Bakkali et al. 2008). A number of EOs are active against *T. cruzi* epimastigotes and trypomastigotes (Santoro et al. 2007a, b, Escobar et al. 2010, Borges et al. 2012, Silva et al. 2013, Azeredo et al. 2014) and recent studies showed that EOs from different pepper species (*Piper* spp.) have promising leishmanicidal (Monzote et al. 2010) and trypanocidal (Esperandim et al. 2013) activities. Nevertheless, the EO of the pepper species *P. aduncum* - whose extracts have clear trypanocidal activity - had not been tested previously against *T. cruzi*.

In the present study, we show that *T. cruzi* trypomastigotes and amastigotes are sensitive to *Pa*EO at concentrations of 2.8 to 12.1 µg/mL (Table I). Importantly, the highly infective cell-derived trypomastigote forms - which can be found in contaminated blood from Chagas disease patients - were particularly sensitive to *Pa*EO, with an IC_50_/24 h of 2.8 µg/mL.

Also, treatment of *T. cruzi* trypomastigotes and human erythrocytes with *Pa*EO at 4ºC resulted in an excellent selectivity index (SI = 92.5) (Table II). The first desirable property of a promising drug candidate is an SI > 50 (Romanha et al. 2010); thus, *P*aEO (or its main constituent linalool) is a promising alternative for further testing on blood/blood cells, under thermal conditions of red blood cells storage (4ºC), in order to obtain derivatives effective at eliminating *T. cruzi* in blood bank samples.

In our tests with cell-derived trypomastigotes at 4ºC the use of BZ for 24 h had no effect against the parasite (not shown). The incomplete trypanocidal effect of BZ at 4ºC can lead to Chagas disease transmission via transfusion of contaminated blood (Martin et al. 2014). Gentian violet was the reference drug of choice in our tests at 4ºC, because it can be used to control *T. cruzi* infection in blood banks (Ramirez et al. 1995). However, the prophylaxis alternative with gentian violet produces side effects, such as staining in the mucosa and erythrocyte agglutination (Docampo & Moreno 1990).

In this study, most testing was performed for 24 h, to minimise the effects of nutrient starvation and toxicity due to parasite lysis. We believe that these conditions are closer to those found in the mammalian host, where toxin waste is eliminated through the bloodstream. Our data with BZ differ from that reported in other studies, where this drug was often tested for 72 h, yielding lower IC_50_ values (Franklim et al. 2013, Hamedt et al. 2014). To date, no standard conditions for drug testing against *T. cruzi* have been defined. Therefore, differences in parasite strains, incubation times and temperatures and culture media may all account for variations observed in experimental drug testing, and are likely to delay drug development for Chagas disease treatment (Andrade et al. 1985).

Mitochondrial membrane potential evaluation of epimastigotes by flow cytometry demonstrated that *Pa*EO decreased the mitochondrial membrane potential of ~98% of the treated cells, with similar results obtained with the positive control with CCCP. Therefore, *Pa*EO may be acting on the parasite mitochondrion. Accordingly, it has been already shown that other natural products induce depolarisation of mitochondrial membrane in Trypanosomatids such as *T. cru*zi and *Leishmania* (Inacio et al. 2012, Caleare et al. 2013, Ribeiro et al. 2013, Takahashi et al. 2013, Aliança et al. 2014, Corpas-López et al. 2016, Sülsen et al. 2016).

EOs may affect different cellular targets, due to variations in their molecular composition. As they contain lipophilic molecules, the mechanism of action of EOs involves breakage and/or crossing of the plasma membrane (Knobloch et al. 1989, Sikkema et al. 1994). While our data showed that the *Pa*EO may target the mitochondrion, we can not exclude the possibility that mitochondrial damage is a secondary effect of drug treatment.

The *Pa*EO used in this work had nerolidol (25.22%) and linalool (13.42%) as its main constituents. GC-MS analysis of the essential oil of *P. aduncum* in other studies showed that it can yield four major constituents: (a) dillapiole, at 79.9-86.9% (Almeida et al. 2009); (b) nerolidol, at 79.2-82.5% (Oliveira et al. 2006); (c) cineole, at 54% (Oliveira et al. 2013), and (d) linalool, at 31-41% (Navickiene et al. 2006). This variation in composition may be explained by the collection of plants from different regions, and which are exposed to different environmental factors. Thus, to minimise EO composition variation between studies, the collected material should always come from the same location. The best thermal storage conditions for *P. aduncum* EO is 20ºC (for up to six months), without loss of regenerative capacity (da Silva & Scherwinski-Pereira 2011).

In this work we show that nerolidol - the major *Pa*EO component - failed to affect significantly the cell-derived trypomastigote form of *T. cruzi*, at 4ºC, even at the concentration of 1 µg/mL. In contrast, linalool had potent trypanocidal effect against this parasite form, with an IC_50_/24 h of 306 ng/mL. These results show that the major component of an EO may not always be responsible for the lytic activity. Interestingly, incubation of linalool with *T. cruzi* blood trypomastigotes (Y strain) resulted in an IC_50_/24 h value of 264 µg/mL (Santoro et al. 2007b), indicating that trypomastigote forms of different origin (blood, cell culture or in vitro differentiation) and from different strains may differ in their susceptibility to EO derivatives.

Our cytotoxicity tests in Vero cells showed that cell monolayers remained intact after nerolidol treatment, without clear changes or toxic effects, even at the concentration of 1 µg/mL. However, linalool was cytotoxic, with CC_50_/24 of 823.6 ng/mL, indicating that the major constituent is not necessarily the one responsible for EO toxicity towards mammalian cells, as suggested in other studies (Almeida et al. 2009, Oliveira et al. 2013, Liu et al. 2014). Also, linalool was approximately 80,000 times more efficient against cell-derived trypomastigotes at 4ºC than gentian violet, the drug commonly used to treat possibly contaminated blood bags.

In conclusion, our data indicate that the *P. aduncum* essential oil component linalool is a promising compound for further studies on the trypanocidal treatment of red blood cell bags at 4ºC prior to transfusion, to prevent *T. cruzi* transmission via this important route. To improve the safety profile of linalool, combinations with less toxic compounds (including benznidazole) should be tested, and linalool could be used as a lead for further drug development.
